# Dividing communication, at the nanoscale

**DOI:** 10.7554/eLife.79446

**Published:** 2022-05-24

**Authors:** Amelia J Ralowicz, Michael B Hoppa

**Affiliations:** 1 Department of Biology, Dartmouth College Hanover United States

**Keywords:** synapses, glutamate release, spontaneous release, evoked release, fluorescence photobleaching, iGluSnFR, Rat

## Abstract

Fluorescent glutamate sensors shed light on the microscopic organization underlining spontaneous neurotransmission.

**Related research article** Wang CS, Chanaday NL, Monteggia LM, Kavalali ET. 2022. Probing the segregation of evoked and spontaneous neurotransmission via photobleaching and recovery of a fluorescent glutamate sensor. *eLife* **11**:e76008. doi: 10.7554/eLife.76008.

Your conscious thoughts, your motivations, your most intricate movements are all built on the computational power that rest within synapses. At these sites, a transmitting presynaptic neuron sends information, in the form of chemical signals known as neurotransmitters, to a receiving postsynaptic cell. Neurotransmission begins when vesicles that store the neurotransmitters fuse with the presynaptic membrane, releasing the molecules into the space between the two neurons. These molecules are then captured by receptors on the membrane of the postsynaptic neuron, altering the activity of the receiving cell.

Most commonly, electrical signals trigger neurotransmitter release through an ‘evoked vesicle fusion’ process. Yet, in 1952 it was discovered that neurotransmission could also occur without electrical activity. This second mode of communication was given the placeholder moniker of ‘spontaneous vesicle fusion’, since its origin and use was unknown (Fatt and Katz, 1952). In fact, this process was originally assumed to be a natural consequence of imprecise molecular interactions. However, more recent evidence suggests that distinct molecular mechanisms underpin spontaneous and evoked vesicle fusion, with the two processes activating separate classes of postsynaptic receptors. This molecular segregation suggests that individual synapses could either specialize in one type of vesicle fusion, or could contain machinery and space for both evoked and spontaneous neurotransmission (Sara et al., 2011; Peled et al., 2014; Cho et al., 2015).

Recent innovations in mathematics and fluorescent microscopy have peered within the half-micron of the synaptic connection, identifying distinct nanometer arrangements of molecules that form a narrow column spanning between both sides of the synapse (Tang et al., 2016). Evoked vesicle fusion and detection takes place inside these columns, with the neurotransmitters being captured by receptors present within a carefully delineated postsynaptic nanodomain (Figure 1). Still, spontaneous fusion events cannot be detected via this approach: therefore, whether evoked and spontaneous vesicle fusion take place in the same location within synapses remains an open question.

**Figure 1.**
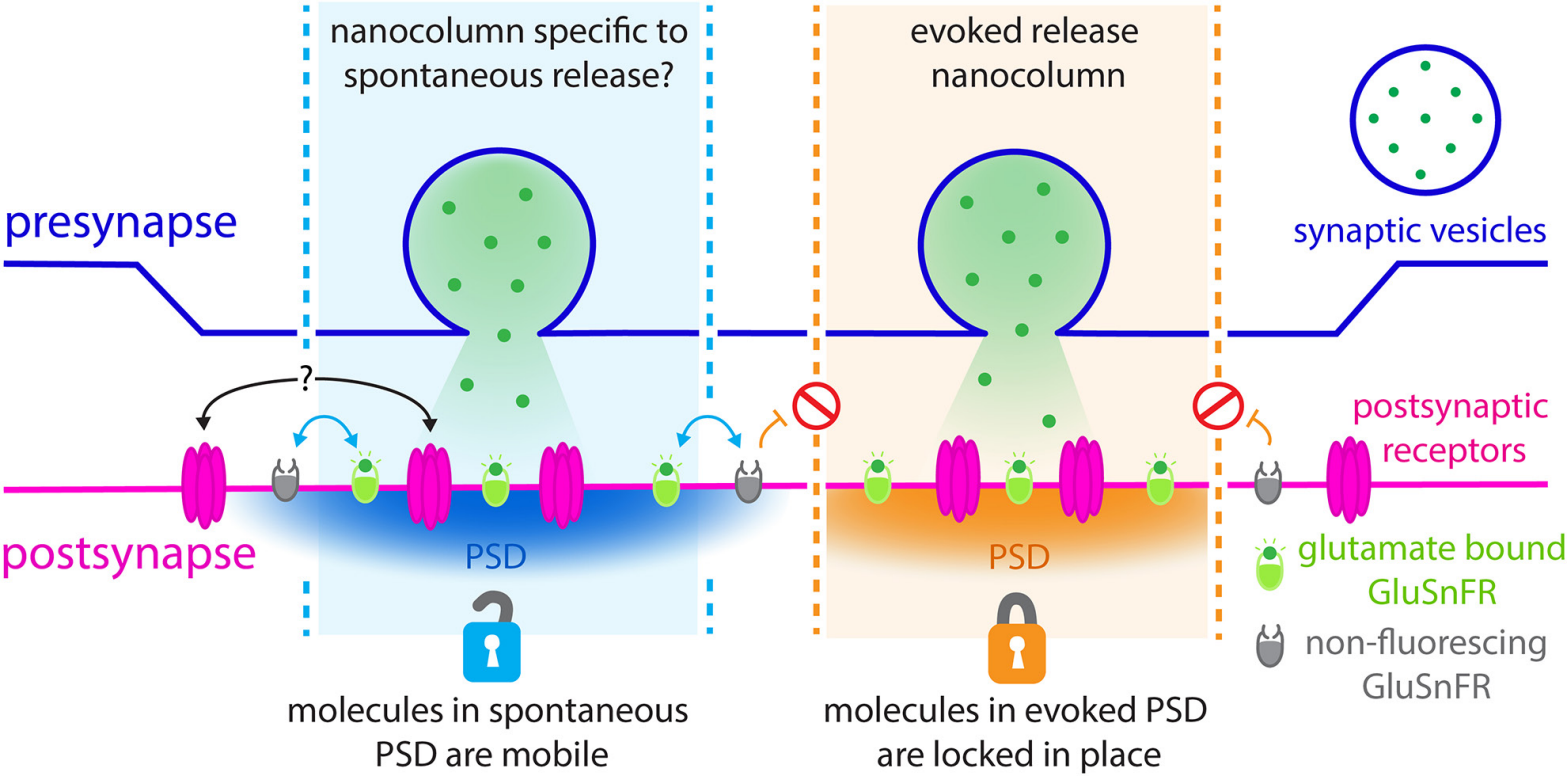
Revealing the nanoscale organisation of synapses during evoked and spontaneous vesicle fusion. Neural information is passed from a presynaptic neuron (top; purple) to a postsynaptic neuron (bottom; pink) through the release of neurotransmitters such as glutamate (green dots). These molecules are stored in the presynaptic neurons inside dedicated vesicles (purple circle) which can fuse with the membrane either spontaneously (left; blue) or evoked by an electric current (right; orange). Glutamate travels across synapses within nanoscale ‘columns’ (pale blue and pale orange) and is captured by receptors (pink) studded through the postsynaptic membrane at dedicated postsynaptic domains (PSD). GluSnFR fluorescent reporters introduced at the postsynaptic membrane, which glow when bound to glutamate (green ovals), can be used to understand the architecture of synapses. To examine what happens during evoked and spontaneous fusion, Wang et al. photobleached the GluSnFRs in the postsynaptic membrane so they could no longer fluoresce (grey ovals). Fluorescence was still observed during spontaneous fusion events, but not during evoked ones. In the postsynaptic domains of spontaneous fusion (left; pale blue column) non-bleached GluSnFRs reporters are mobile and can diffuse (blue arrows) in to these locations to replace the bleached reporters. It is possible that the actual postsynaptic receptors also do this (black arrows with question marks). However, this diffusion does not take place (orange arrows and red signs) in the postsynaptic domains of evoked fusion (pale orange column). Different rules of diffusion for GluSnFrs therefore exist within these segregated sites of neurotransmission.

An emerging method in the field of neuroscience involves inserting fluorescent reporters into the postsynaptic membrane: these reporters glow when bound to the neurotransmitter glutamate, thus allowing scientists to visualize where and when neurotransmission occurs in the brain. However, these GluSnFR molecules are likely to ‘photobleach’, becoming unable to fluoresce if exposed to too much light stimulation. Now, in eLife, Ege Kavalali and colleagues at Vanderbilt University – including Camilla Wang as first author – report having used this perceived drawback of GluSnFRs to track where spontaneous vesicle fusion occurs (Wang et al., 2022).

From a methodology standpoint, the team established a new protocol to measure spontaneous vesicle fusion with unprecedented reliability. Previously, scientists could tell when spontaneous neurotransmission was taking place by measuring the electrical activity of receiving neurons, but the technique could not determine where this process was located among thousands of synapses. Wang et al. established protocols for using glutamate sensors to tell both where and when spontaneous vesicle fusion occurs. This is a thrilling development for future experiments, especially as more stable and quantitative versions of GluSnFRs are developed to measure how much neurotransmitter is released across longer measurement times.

From a scientific standpoint, Wang et al. made a breakthrough by leaning into the high bleaching rates of the current glutamate sensor. First, neurons were electrically stimulated, and successful evoked and spontaneous events were measured at individual synapses. Then, all GluSnFRs present in the microscope’s field of view were photobleached, meaning that any emerging fluorescence would signal the presence of non-photobleached GluSnFRs migrating from other locations within the postsynaptic membrane. Spontaneous neurotransmission could still be detected under these conditions, but evoked glutamate release could not be spotted for close to an hour (Figure 1).

Paired with numerous controls, these experiments establish that most synapses support both types of fusion. In addition, they imply that these events take place at distinct locations within synapses, with GluSnFRs moving within the membrane differently within evoked and spontaneous postsynaptic nanodomains. In particular, a large fraction of GluSnFRs seems to be immobile at the postsynaptic membrane during evoked fusion, as suggested by the absence of non-photobleached reporters diffusing to these sites to replace the deactivated GluSnFRs.

Taken together, the results demonstrate that evoked and spontaneous vesicle fusion occur in discrete nanoscale postsynaptic locations, each featuring different diffusion characteristic for GluSnFRs – and, potentially, for actual postsynaptic glutamate receptors. This is further supported by prior studies which have also identified a mobile and immobile fraction of these proteins at the synapse (Heine et al., 2008; Graves et al., 2021).

Overall, the work by Wang et al. helps to fuel an intense field of study which has started to explore how brain activity and neural pathologies are rooted in the nanoscale organization of synapses (Ramsey et al., 2021; Hruska et al., 2022). More excitingly, it also offers a new experimental tool to begin investigating the neural function of spontaneous vesicle fusion.
